# Current and emerging therapeutic strategies for amyotrophic lateral sclerosis: from pharmacological approaches to gene and stem cell therapies

**DOI:** 10.3389/fneur.2026.1729302

**Published:** 2026-01-22

**Authors:** Ze Wang, Jiajun Huang, Di Yun

**Affiliations:** 1 Department of Pharmacy, The Second People's Hospital of Neijiang, Neijiang, Sichuan, China; 2 Department of Neurology, The Second People's Hospital of Neijiang, Neijiang, Sichuan, China; 3 School of Life Science and Technology, Shanghai Tech University, Shanghai, China

**Keywords:** amyotrophic lateral sclerosis (ALS), gene therapy, pharmacological treatments, potential therapeutic targets, stem cell therapy

## Abstract

Amyotrophic lateral sclerosis (ALS) is a progressive neurodegenerative disease that involves upper and lower motor neurons, severely impairing patients’ quality of life. The complex interaction of genetic and environmental factors in ALS pathophysiology complicates therapeutic development. Currently available disease-modifying pharmacological therapies for ALS offer limited efficacy, only slowing disease progression to a modest degree. The recent market withdrawal of a previously approved therapy (AMX0035) further underscores the challenges in this field. Biological targets for ALS and related neurodegenerative diseases offer a unique avenue for therapeutic intervention. With the advancement of genetic engineering technology, innovative therapies such as Stem cell therapy and gene therapy are also discussed, offering a promising horizon for ALS treatment. In addition, the management of ALS symptoms plays a key role in improving the daily lives of people with the disease. In this review, we summarize various strategies for treating ALS, providing an overview of the disease.

## 1 Introduction

Amyotrophic lateral sclerosis (ALS) is a fatal neurodegenerative disease characterized by the progressive loss of upper and lower motor neurons, leading to muscle weakness, paralysis, and ultimately respiratory failure (1–3). With a typical survival of 2–5 years from symptom onset, ALS imposes a devastating burden on patients (4). Therapeutic options remain severely limited. Riluzole and edaravone, the long-standing standards of care, offer only modest slowing of disease progression (5, 6)^24^. The recent market withdrawal of AMX0035 following its Phase 3 trial failure further underscores the profound challenges in ALS drug development (7). The accelerated approval of tofersen for SOD1-ALS marks a pivotal advance in precision medicine but also highlights a critical gap: effective strategies for the vast majority of patients without such targetable mutations (8, 9).

A critical consideration in ALS research and therapy development is the distinction between familial (fALS) and sporadic (sALS) forms. While fALS, accounting for approximately 5–10% of cases, is defined by identifiable genetic mutations (e.g., in SOD1, C9orf72), sALS constitutes the overwhelming majority (~90%) (10). The etiology of sALS is complex and multifactorial, thought to arise from a combination of genetic susceptibility, environmental exposures (e.g., certain toxins, viral infections), age-related alterations, and epigenetic modifications (11). This profound etiological heterogeneity presents a fundamental challenge for developing broadly effective treatments.

This heterogeneity necessitates a multi-pronged therapeutic strategy. Current research spans pharmacological neuroprotection, modulation of convergent pathological pathways (e.g., TDP-43 proteinopathy, neuroinflammation) common to both fALS and sALS (12–14), and innovative biological approaches like stem cell and gene therapy (15, 16). This review aims to provide a focused overview of this evolving landscape. We critically summarize the mechanisms, efficacy, and limitations of approved and emerging pharmacological treatments. Furthermore, we analyze the rationale, current progress, and challenges of stem cell and gene therapies, with particular emphasis on the imperative to address sporadic ALS. Finally, we underscore the indispensable role of comprehensive symptomatic management in patient care (17).

## 2 Advances in pharmacological treatments for ALS

Advancements in the pharmacological landscape for ALS have introduced a handful of disease-modifying treatments. At present, there are three drugs including riluzole, edaravone and AMX0035 have been approved by FDA (Table 1). It is worth noting that the results of the Phase 3 PHOENIX trial of AMX0035 were not satisfactory, and the manufacturer has initiated a market withdrawal procedure. Moreover, tofersen has gained accelerated approval, pending further substantiation of its clinical efficacy in ongoing trials. Customizing therapeutic approaches to align with individual patient profiles is essential, especially given that existing guidelines have not incorporated the latest approvals, such as tofersen (8).

**Table 1:** The difference of clinical trial drugs.

Drug Name	Tofersen	Riluzole	Edaravone	AMX0035	Nuedexta
Molecular Formula	Not specified	C₈H₅F₃N₂OS	C₁₀H₁₀N₂O	Not specified	C_18_H_26_BrNO / C_20_H_24_N_2_O_2_
Mechanism of Action	ASO mediating degradation of SOD1 mRNA.	Glutamate release inhibitor; reduces excitotoxicity.	Free radical scavenger; antioxidant.	Targets ER and mitochondrial stress pathways.	Sigma-1 receptor agonist, NMDA receptor antagonist.
Target	SOD1 mRNA	Voltage-gated sodium channels; Glutamate.	Free radicals	ER stress; Mitochondrial dysfunction.	Sigma-1 receptor; NMDA receptor; CYP2D6 enzyme.
Drug Type	Antisense oligonucleotide	Small molecule	Small molecule	Oral fixed-dose combination	Fixed-dose combination (Dextromethorphan/Quinidine)
Approval date (US FDA)	2005	1995	2017	2022	2010
Clinical Trials Phases	Phase III	Pivotal Phase III	Phase III	Phase II/III	Phase III for PBA in ALS/MS; Phase II for bulbar function in ALS.
Company developed	Ionis Pharmaceuticals and Biogen	Sanofi (Rilutek)	Mitsubishi Tanabe (Radicava)	Amylyx Pharmaceuticals	Avanir Pharmaceuticals (now part of Otsuka)
Efficacy in ALS	Reduced SOD1 protein levels and NfL levels	Modestly prolongs survival (by 2–3 months).	Slows rate of functional decline.	Slows functional decline (ALSFRS-R score); long-term follow-up showed extended median survival.	Approved for Pseudobulbar Affect (PBA) in ALS. Recent evidence suggests off-label use may improve bulbar function (speech, swallowing).
Side Effects	Pain, fatigue, joint pain, increased CSF white blood cells, myalgia	Headache, abdominal pain, back pain, vomiting, dyspepsia, diarrhea, dizziness.	Bruising, gait disturbance, headache.	Gastrointestinal events (e.g., diarrhea, nausea).	Diarrhea, dizziness, cough, vomiting, peripheral edema, urinary tract infection.

### 2.1 Mechanism of action and efficacy of drugs

#### 2.1.1 Riluzole

**Figure 1:**
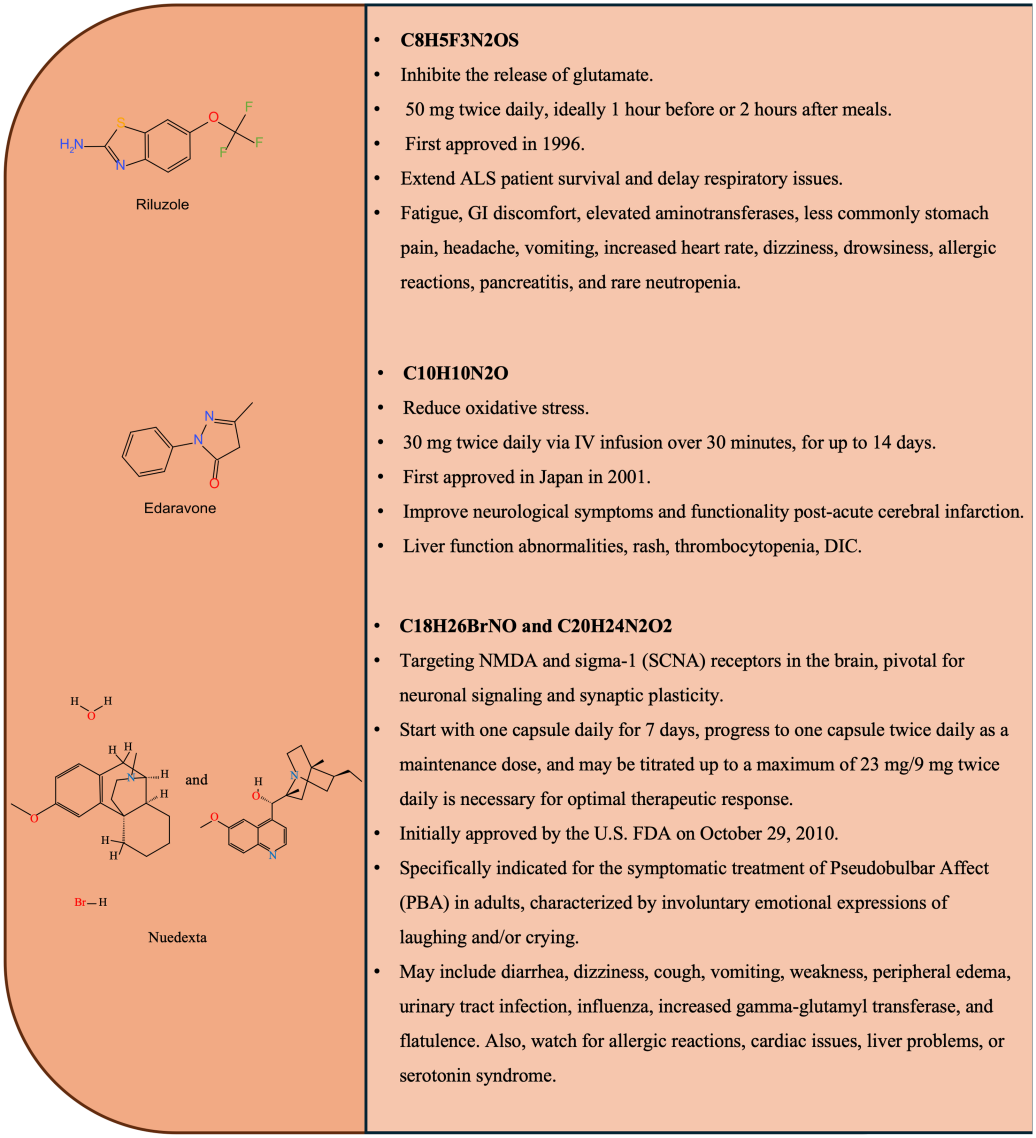
Chemical structures and key features of clinical trial drugs.

As the first FDA-approved drug for ALS (1995), riluzole has been the cornerstone of pharmacological management for decades. Then, Riluzole was approved for the treatment of ALS in most countries (18, 19). Although many other drugs have been studied, it was the only clinically approved treatment for ALS for more than two decades. The chemical structures and key features of riluzole are shown in Figure 1. In ALS, glutamate homeostasis is dysregulated and glutamate-mediated excitotoxicity is regarded as the key mechanism of ALS pathogenesis (20). As a benzothiazole drug, the drug operates on a foundation of neuronal protection, blocking voltage-gated sodium channels to reduce the release of the excitatory neurotransmitter glutamate and increasing glutamate uptake through excitatory amino acid transporter to regulate extracellular glutamate levels (21). This action regulates subsequent intracellular activities post-neurotransmitter binding, and curbs excitotoxicity, thus protecting neurons from damage. Riluzole regulates intracellular Ca^2+^, thereby maintaining calcium homeostasis. In addition, it increases oxidative stress and interferes with integrity of DNA, as well as autophagic and apoptotic pathways (22).

Riluzole is more effective in patients with advanced disease, although it is recommended for patients with all stages of ALS (23, 24). Evidence from clinical trials has validated riluzole’s effectiveness in extending median survival of ALS patients by two to three months and slowing the decline in respiratory capabilities, a significant marker of ALS progression (25). They also indicated that riluzole could positively affect motor functions (25). Real-world investigations from Italy also indicated that riluzole treatment reduced the mortality rate of ALS patients (26). In all the studies, patients demonstrated high adherence to treatment and good drug tolerance. Its use requires vigilance for adverse effects, most notably potential hepatotoxicity, which necessitates regular monitoring of liver function. Despite its long-standing status, the clinical benefits of riluzole are modest. It extends median survival by only approximately 2 to 3 months, a benefit that many patients and clinicians consider limited in the face of a rapidly progressive disease (27). Furthermore, real-world evidence regarding its effectiveness remains mixed. Some observational studies have reported no significant improvement in survival or functional decline, highlighting the discrepancy that can exist between controlled clinical trials and broader clinical practice (28). Safety monitoring is also required, as post-marketing surveillance indicates a need for vigilance regarding adverse events such as interstitial lung disease, hepatic dysfunction, and pancreatitis (5). However, there are also surveys indicating that riluzole neither improved survival nor slowed functional decline in ALS patients (28). These contradictory clinical investigation results indicate the limited efficacy of the drug. The evidence is still insufficient to draw any definite conclusion and more extensive research is needed.

#### 2.1.2 Edaravone

Edaravone, approved in Japan (2015) and the US (2017), represents another major pharmacological option, particularly indicated for early-stage ALS (23). Its chemical structures and key features are shown in Figure 1. The full extent of its therapeutic effects in ALS is still being under comprehensive exploration. Oxidative stress is considered to be involved in the pathology of ALS. Edaravone exhibits strong biological activities including inhibition of oxidative stress and scavenging free radicals (29). Its antioxidant mechanism is central to its efficacy, skillfully countering the pernicious reactive oxygen species (ROS) that initiate lipid peroxidation, although its specific mechanism of action is still unknown (30). Studies have shown that edaravone reduces the accumulation of hydrogen peroxide (H_2_O_2_) through upregulation of Peroxiredoxin-2 (31), and can also trap hydroxyl radicals (^. OH^) and peroxynitrite anions (ONOO^−^) in its anionic form (30). Furthermore, the drug’s impact is notably robust in the context of cerebral ischemia and reperfusion, adeptly mitigating oxidative stress, brain edema, and the resultant tissue damage by inhibiting NADPH oxidase 2 (NOX2) (32).

A 2.5-year retrospective study from India suggests that intravenous edaravone treatment has no beneficial effect on the Amyotrophic Lateral Sclerosis Functional Rating Scale (ALS-FRS) score and does not improve survival rate (33). Another observational study from India shows that edaravone infusion does not stop or significantly slow progression of ALS from baseline but is safe (34). On the contrary, clinical trials from the United States have shown that edaravone oral suspension can significantly prolong survival time and reduce the decline of physical functions (35). These recent clinical investigation results all suggest that edaravone has limited efficacy in the treatment of ALS. In addition, the application of edaravone has been expanded to include acute ischemic stroke, with a Phase III clinical trial underscoring the enhanced efficacy of edaravone when combined with dexborneol, suggesting the merits of this combined approach (36). The approval of edaravone for both ALS and stroke therapy signifies a significant leap forward in neuroprotective strategies. Considering its capacity to target oxidative stress, a pervasive factor in numerous neurological disorders, edaravone is poised to extend its relevance beyond its current indications. Upcoming studies may reveal additional therapeutic potential for edaravone, including its synergistic interactions with other compounds or its utility in addressing a wider spectrum of neurological diseases. With ongoing clinical investigations, edaravone holds the promise of refining patient outcomes and enriching the array of options within the neurological treatment domain. Important safety considerations include the risk of hypersensitivity reactions and the need for renal function monitoring during treatment. The efficacy evidence for edaravone is primarily derived from clinical trials in East Asian populations, and its benefits have been less consistently replicated in Western studies and real-world settings outside of Japan. This discrepancy may be attributed to disease heterogeneity, differences in patient enrollment criteria (e.g., disease stage and progression rate), or genetic factors (37). A real-world study from India suggested that edaravone infusion did not significantly alter disease progression (34). While generally safe, post-marketing data analysis indicates that adverse events related to edaravone, though mostly non-severe, do occur and necessitate clinical awareness (38, 39). These factors collectively suggest that edaravone’s therapeutic effect may be most pronounced in a specific subset of ALS patients, and its global applicability requires further careful evaluation.

#### 2.1.3 AMX0035

AMX0035 (sodium phenylbutyrate and taurursodiol) received conditional approval in 2022 based on early-phase data, representing a novel dual-pathway targeting approach. The safety and tolerability of AMX0035 had been previously confirmed in small PALS trials. The phase 2 CENTAUR study and its open-label extension demonstrated the safety and efficacy of AMX0035 in PALS (7). AMX0035 had shown significant slowing of disease progression and prolonged survival (40). It is worth noting that Amylyx Pharmaceuticals announced the results of the phase 3 PHOENIX trial of AMX0035 for the treatment of ALS in 2024. The primary endpoint or secondary endpoints did not reach statistical significance as measured by change from baseline in the Revised ALSFRS (ALSFRS-R) or ALSAQ-40 and SVC, despite with good tolerance and safety. Survival data will continue to be collected. The journey of AMX0035 underscores the critical challenge of replicating early positive signals in larger, confirmatory trials. While the phase 2 CENTAUR trial showed a slowing of functional decline, the subsequent phase 3 PHOENIX trial failed to meet its primary or secondary efficacy endpoints, leading to the drug’s voluntary withdrawal from the market (7). Several hypotheses may explain this discrepancy. The PHOENIX trial had a longer placebo-controlled period (48 vs. 24 weeks) and a more geographically diverse population, which may have introduced greater clinical variability (41). Differences in baseline patient characteristics, including a lower concurrent use of other ALS therapies in PHOENIX compared to CENTAUR, might also have influenced outcomes. An expert commentary suggested that the positive result in the smaller CENTAUR trial could have been a false positive, or that the treatment effect is too small to be reliably detected without highly homogeneous patient groups (42). This case highlights the risks of accelerated approvals based on single, modest-sized trials and the imperative for robust phase 3 validation. Amylyx has announced that it has started a process with the FDA and Health Canada to voluntarily discontinue the marketing authorizations for AMX0035 based on topline results from the Phase 3 PHOENIX trial. The therapy was generally well-tolerated, with gastrointestinal events such as diarrhea and abdominal pain being the most frequently reported adverse effects. The limited efficacy of the currently approved clinical drugs highlights the need for the study and development of new drugs.

#### 2.1.4 Tofersen

Tofersen, crafted by Biogen as an innovative antisense oligonucleotide therapy, has a specific indication for adults with ALS who have a mutation in the SOD1 gene. This precision medicine disarms the disease at the genetic level by hybridizing with SOD1 mRNA, orchestrating its degradation and consequently snuffing out the production of the detrimental SOD1 protein (8, 43, 44). Intrathecal administration ensures that tofersen targets motor neurons with pinpoint accuracy through cerebrospinal fluid, effectively diminishing cerebrospinal levels of SOD1 protein and plasma levels of neurofilament light chains—biomarkers that signal the regression of neuronal damage (43).

Although the VALOR trial did not meet its primary milestone, tofersen’s ability to significantly reduce these biomarkers was not overlooked, earning it an expedited FDA approval on April, 2023 (9). Marking its place as the fourth ALS treatment and pioneering the way as the inaugural gene therapy to gain accelerated approval anchored in biomarker evidence, tofersen is a landmark in the field of gene therapy for ALS (45). It extends a beacon of hope and a novel therapeutic avenue to improve the medical prognosis and quality of life for people living with SOD1 mutation-associated ALS. Recent analyses highlight that tofersen not only validates the genetic approach but also enhances therapeutic opportunities by potentially altering the disease course when initiated early, though accessibility and long-term management strategies remain areas for development (46). Serious adverse events associated with its intrathecal administration include myelitis/radiculitis and elevations in intracranial pressure, requiring careful clinical monitoring. The approval of tofersen represents a landmark in precision medicine for ALS but also introduces a nuanced paradigm for evaluating efficacy. Crucially, the pivotal VALOR phase 3 trial did not meet its primary clinical endpoint (change in ALSFRS-R score at 28 weeks) (44). Its accelerated approval was primarily based on a compelling reduction in plasma neurofilament light chain (NfL), a biomarker of neuronal damage, which decreased by approximately 60% in the tofersen group compared to 20% in the placebo group (44). This dissociation underscores the principle that biomarker improvement does not equate to immediate, measurable clinical benefit, suggesting a potential delay between biological effect and functional stabilization (47). The ongoing ATLAS study in presymptomatic SOD1 carriers may provide further insights into whether early intervention can delay clinical onset. Thus, tofersen illustrates both the promise of targeted genetic therapy and the current reality that its most significant impact may be on disease biology, with clinical benefits requiring longer timeframes to manifest or being more modest than initially hoped.

#### 2.1.5 Nuedexta

Nuedexta, a synergistic blend of dextromethorphan hydrobromide and quinidine sulfate, has received FDA approval for the treatment of pseudobulbar affect (PBA) in ALS patients in 2010 (48). The formulation’s efficacy is based on dextromethorphan’s effect on neurotransmission, which can mitigate excitotoxic effects, coupled with quinidine’s ability to enhance dextromethorphan’s cerebral presence by inhibiting efflux transporter activity (49). This pharmacological tandem enhances Nuedexta’s ability to stabilise the neural messengers that regulate emotional responses, thereby alleviating the distress of PBA.

Expanding its therapeutic horizons, Nuedexta is being investigated for its benefits in ALS, where it may provide neuroprotection by targeting the sigma-1 receptor and limiting glutamate-induced excitotoxicity (50). Preliminary clinical evidence suggests that Nuedexta may support bulbar functions critical to speech, swallowing and breathing in ALS, while managing a spectrum of manageable side effects (51). As the understanding of Nuedexta, it may emerge as a multi-faceted therapeutic contender, able to address a range of emotional and motor challenges across the spectrum of neurodegenerative diseases.

### 2.2 Potential therapeutic targets

Researchers are delving into a spectrum of biological targets to combat ALS and related neurodegenerative diseases, each offering a unique avenue for therapeutic intervention. There are numerous potential therapeutic targets, such as the development of targeted inhibitors of ion channels (sodium channels, potassium channels, calcium channels, etc.) and glutamate receptors, to reduce the neuronal excitotoxicity caused by excessive glutamate release (52, 53). Neuroinflammation is a major cause of ALS and other neurodegenerative diseases; by targeting pro-inflammatory factors and reducing the abnormal activation of microglia, the damage caused by an overactive immune response in the central nervous system to neuronal cells can be mitigated (13). Excessive oxidative stress can cause irreversible damage to neuronal cells; enhancing the activity of antioxidant enzymes such as superoxide dismutase (SOD) and glutathione peroxidase (GPx) can reduce oxidative stress levels, potentially leading to the alleviation or cure of ALS (54). In addition, high levels of misfolded protein aggregates are often found in the bodies of ALS patients; these aggregates not only disrupt cellular homeostasis but also trigger apoptotic pathways leading to massive neuronal cell death. Activating the autophagy-lysosome pathway can clear these misfolded protein aggregates, or overexpressing quality control factors in cells, such as molecular chaperones, can help correct the misfolded proteins (55). It is also possible to increase the expression of neuroprotective factors in the body through gene therapy or drugs, such as Brain-Derived Neurotrophic Factor (BDNF) and Glial Cell Line-Derived Neurotrophic Factor (GDNF), thereby enhancing the survival ability of neuronal cells and extending the lifespan of ALS patients (56). Furthermore, the occurrence and development of ALS and other neurodegenerative diseases are also closely related to mitochondrial dysfunction; improving mitochondrial function or regulating metabolic pathways can enhance the energy supply and survival capacity of neuronal cells (57). Given that targeted genetic therapies are primarily applicable to the minority fALS cases, a major strategic imperative is to develop interventions for the overarching pathological processes common to both fALS and sALS. Among these, TDP-43 proteinopathy, characterized by its mislocalization and aggregation in the cytoplasm, is observed in over 95% of all ALS cases, making it a prime target for sALS (12). Other convergent mechanisms offering therapeutic avenues for the sporadic majority include sustained neuroinflammation, persistent oxidative stress, and mitochondrial dysfunction (14). Advancing therapies against these shared pathological nodes, rather than specific mutations, is essential for creating impactful treatments for the vast sALS population. A number of drugs targeting these potential therapeutic points are in clinical trials or in development, offering new hope for ALS drug development and the alleviation and cure of the disease.

## 3 Innovative therapies for ALS

### 3.1 Stem cell therapy for ALS

**Figure 2:**
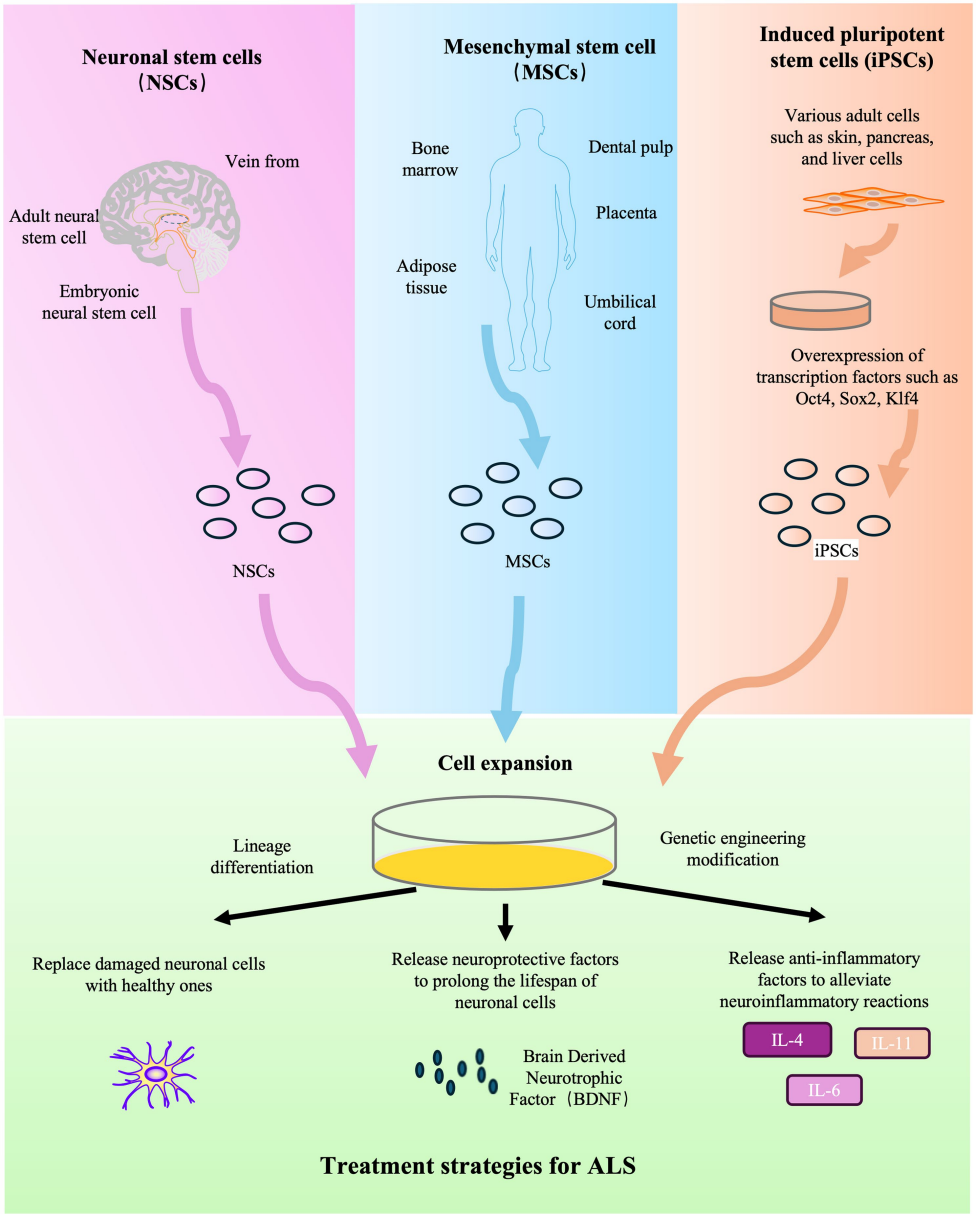
Schematic diagram of stem cell therapy for ALS.

Stem cell therapy is an emerging frontier in the treatment of ALS, aiming to provide neuroprotection, modulate neuroinflammation, and potentially restore damaged motor neurons (Figure 2). This strategy leverages various cell types, including mesenchymal stem cells (MSCs), neural stem cells (NSCs), and induced pluripotent stem cells (iPSCs).

#### 3.1.1 Preclinical foundations and mechanisms

Preclinical studies in animal models have established the foundational rationale for this approach. Research utilizing models such as the SOD1 G93A transgenic mouse has demonstrated that stem cell transplantation can attenuate neuroinflammation and provide trophic support. For instance, MSC transplantation in these models has been shown to modulate the neuroinflammatory milieu by suppressing the activation of detrimental immune cells (58). Furthermore, stem cells offer a platform for delivering neurotrophic factors; for example, neural progenitor cells engineered to secrete glial cell line-derived neurotrophic factor (GDNF) have been explored in preclinical settings (59). These investigations suggest that benefits may arise not only from potential cell replacement but also through powerful paracrine effects. However, challenges such as the long-term survival and integration of transplanted cells within the hostile ALS microenvironment remain significant hurdles in preclinical models (60, 61). The combination of biomaterials and stem cells is also being investigated as a new approach in preclinical research (62). Beyond direct therapy, stem cells are deployed to model ALS *in vitro*, providing insights into disease mechanisms and revealing new therapeutic targets (63).

#### 3.1.2 Clinical translation: trials and challenges

Translation to human trials has progressed through several phases, with early-phase studies primarily assessing safety and feasibility. A phase 1/2a trial demonstrated that the transplantation of human neural progenitor cells secreting GDNF (CNS10-NPC-GDNF) into the spinal cord was feasible and safe over 42 months, with no negative effects on motor function (64). Another pivotal phase 3 trial evaluated MSCs induced to secrete neurotrophic factors (MSC-NTF, NurOwn). While the study reported positive effects on cerebrospinal fluid biomarkers of neuroinflammation and neurodegeneration, it did not meet its primary clinical efficacy endpoint, highlighting the difficulty of translating biological signals into measurable clinical benefits in a heterogeneous patient population (65). These clinical trials are diligently assessing the safety, feasibility, and efficacy of various stem cell types and transplantation strategies (64, 65). The common challenges faced in clinical translation include immune rejection, the variability in patient responses, and the need for standardized cell products and delivery protocols. The decisive impact on disease trajectory and survival rates remains under investigation, underscoring the need for further rigorously designed clinical studies.

### 3.2 Gene therapy for ALS

**Figure 3:**
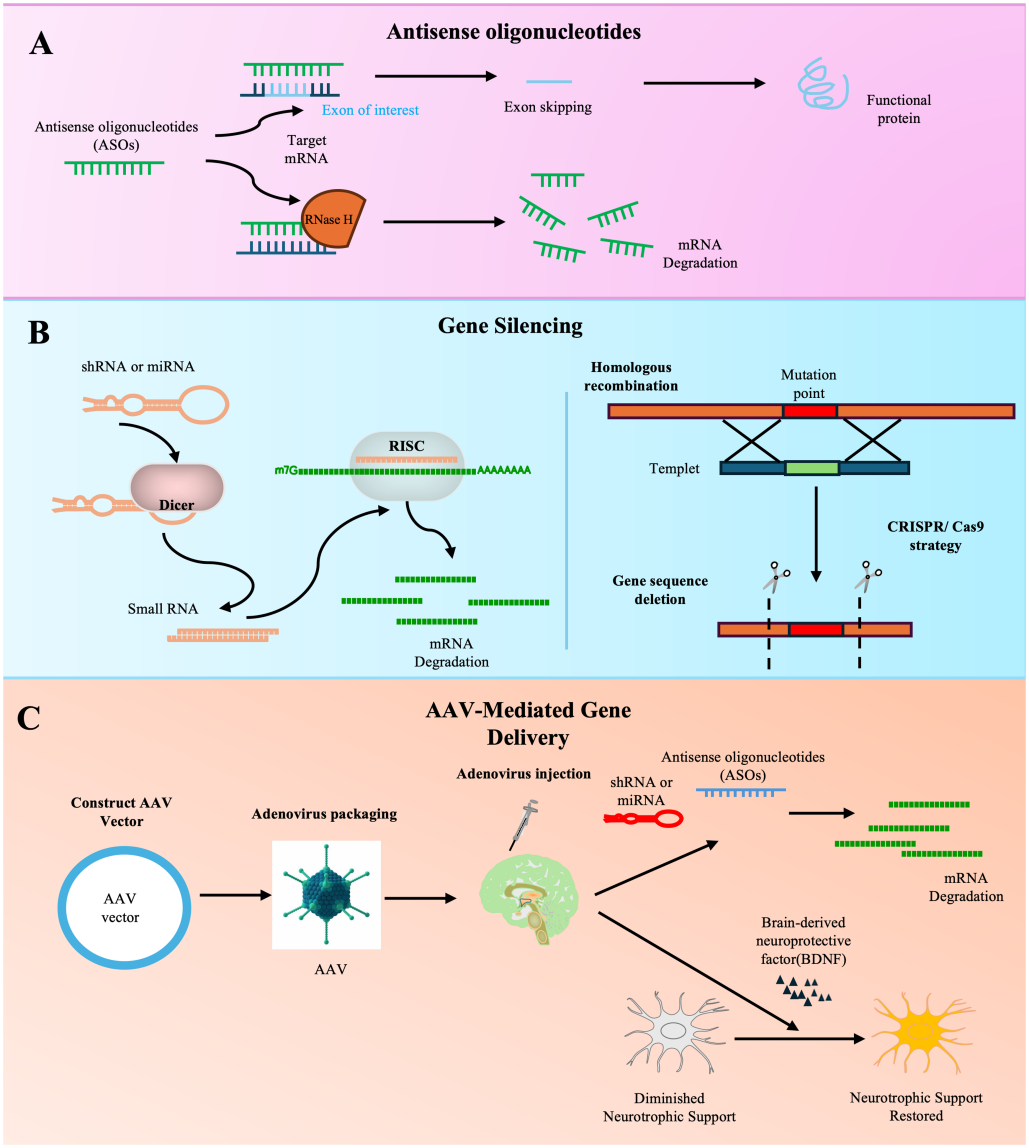
Schematic illustration of gene therapy approaches for ALS.

Gene therapy is emerging as a beacon of hope in the search for transformative treatments for ALS, a disease marked by its relentless progression and complex genetic architecture (Figure 3). Innovative strategies include gene silencing, editing, and the delivery of neurotrophic factors.

The most advanced clinical progress has been made with antisense oligonucleotides (ASOs). Clinical trials have established the proof-of-concept for intrathecal ASO therapy, exemplified by tofersen for SOD1-ALS, which achieved accelerated approval based on biomarker reduction despite not meeting the primary clinical endpoint in its phase 3 VALOR trial (8, 43, 44). Ongoing clinical trials are evaluating other ASO candidates targeting genes such as C9orf72 (e.g., WVE-004, NCT04931862) and FUS (e.g., ION363, NCT04768972), as well as ASOs for other targets like ATXN2 (BIIB105, NCT04494256) and SOD1 (e.g., ISIS SOD1Rx, NCT01041222) (8, 15, 16).

Concurrently, substantial preclinical research is exploring other delivery platforms and strategies. Studies in animal models, particularly the SOD1 G93A mouse, have shown that silencing mutant SOD1 via ASOs or other means can be effective (66). Preclinical work has also confirmed that adeno-associated virus (AAV)-mediated delivery, such as spinal subpial delivery of AAV9, enables widespread gene silencing and can block motor neuron degeneration in rodent models of ALS (67). Furthermore, preclinical studies are investigating strategies to correct downstream pathologies common in sporadic ALS, such as targeting TDP-43 proteinopathy. For example, ASOs designed to correct TDP-43-dependent STMN2 cryptic splicing have shown promise in preclinical models (68). Research also explores AAV-mediated delivery of neurotrophic factors (NTFs) or modulators of neuromuscular junctions (NMJs) to support motor neuron survival (16).

Despite the optimism, the field must navigate significant challenges. These include devising efficient delivery systems to target the brain and spinal cord broadly, ensuring the precision of gene editing to avoid off-target effects, and addressing ethical considerations. Critically, the predominant sporadic form of ALS (~90% of cases) lacks defined genetic targets, complicating patient stratification and demanding the development of therapies targeting convergent pathological pathways (15, 16). A synergistic, multitargeted approach may eventually transform this devastating disease into a controllable chronic one.

### 3.3 Challenges and future directions

The translation of stem cell and gene therapies from promise to practice faces a spectrum of interconnected challenges. Technical and biological hurdles are paramount, including the inefficient delivery of cells or vectors to widespread motor neurons across the central nervous system, host immune responses that can limit efficacy or durability, and unresolved long-term safety concerns such as off-target effects or uncontrolled cell differentiation (15, 60, 65).

Furthermore, significant clinical and translational barriers must be overcome. These include the prohibitive costs and complex manufacturing of therapies, which threaten equitable access; evolving ethical and regulatory frameworks for evaluating these advanced interventions; and, most critically, the “target dilemma” posed by sporadic ALS (~90% of cases). The lack of defined genetic drivers in most patients limits the immediate applicability of precision gene-silencing approaches, necessitating a shift towards targeting convergent pathological pathways and developing biomarkers for patient stratification. Innovations in drug delivery systems, such as advanced nanocarriers and novel intrathecal formulations, are actively being explored to overcome the biodistribution and bioavailability barriers specific to the CNS, which are crucial for both biological therapeutics and small molecules (69). A clear-eyed acknowledgment of these challenges is essential to guide the focused research and collaborative innovation needed to realize the transformative potential of these therapies.

## 4 Management of ALS symptoms

Beyond these disease-slowing strategies, the management of ALS symptoms plays a pivotal role in enhancing the day-to-day living experience for those affected (17). Despite a dearth of comprehensive evidence for numerous pharmacological interventions, healthcare providers have turned to symptomatic treatments to mitigate a spectrum of issues, including psychological distress like anxiety and depression, emotional volatility (pseudobulbar affect), involuntary muscle twitches, pervasive fatigue, sleep disturbances, and a range of physical discomforts from muscle cramps and spasms to immobility-induced musculoskeletal pain, neuropathic pain, sialorrhea, spasticity, constipation, and urinary urgencies.

Respiratory failure is a significant cause of morbidity and mortality in patients with ALS. Noninvasive ventilation is proved to improve several measures of quality of life and extend survival in patients with ALS on average 205 days in clinical trials (70). Another clinical study also showed that underutilization of noninvasive ventilation could influence survival outcomes in patients with ALS (71). Effective airway clearance is the key to clinical care and a mild-intensity respiratory strength training program can improve maximum expiratory pressure in patients with early-stage ALS (72). Pay particular attention to dysphagia and weight loss during nursing. Enteral nutrition is the appropriate intervention when patients have lost more than 10% of their premorbid bodyweight (73). Another thing that needs to be considered for ALS patients is muscle spasticity. The most common antispasticity medication in clinic is baclofen (74).

Though these interventions may not offer a cure, they are masterfully orchestrated to complement medical treatments, sensitively attuned to the individualized and dynamic requirements of each ALS patient. The collective goal is to elevate the patient’s quality of life at every turn of the disease’s trajectory, while ensuring that caregivers are encircled with the support and resources required to deliver the most empathetic and effective care.

## 5 Discussion

Numerous comprehensive reviews have effectively outlined the pathophysiology and therapeutic pipeline of ALS. The distinctive value of the present review lies in its timely and critical analysis of the field’s shifting paradigm, informed by developments from 2023–2025. We extend beyond cataloging drugs by contextualizing landmark events such as the first biomarker-driven accelerated approval (tofersen) and a major Phase 3 trial failure leading to market withdrawal (AMX0035) to discuss their implications for clinical trial design and therapeutic strategy. Furthermore, we place significant emphasis on the formidable challenge of treating sporadic ALS, dedicating analysis to emerging, non-mutation-specific targets and delivery technologies. In contrast to a purely descriptive approach, this review strives to synthesize these elements to illustrate the ongoing transition from broad neuroprotection towards a dual strategy of precision medicine for defined subgroups and mechanism-based combination therapies for a broader patient population.

The therapeutic vista for ALS is in a state of dynamic evolution, necessitating a multi-pronged strategy to confront the intricate and variable nature of the disease. Pharmaceutical innovations like Edaravone, Riluzole and Tofersen, have made inroads against the disease’s progression and approved by FDA (19, 23). Nuedexta has also received FDA approval for the treatment of pseudobulbar affect (PBA) in ALS patients (48). However, AMX0035, which has been approved by the FDA, has started a process to voluntarily discontinue the marketing authorizations based on topline results from the Phase 3 PHOENIX trial. The limited efficacy of the currently approved clinical drugs underscores an urgent call for the study and development of new drugs and innovative therapies for ALS. Stem cell therapy, with its observed efficacy in preclinical settings, beckons with the promise of neuronal regeneration, yet it grapples with the complex challenges of cellular integration, immune reactions, and ethical quandaries. The dawn of gene therapy, exemplified by Tofersen, heralds a pivotal shift towards precision medicine for genetic subtypes. However, this success starkly highlights the “target dilemma” for the approximately 90% of patients with sporadic ALS, where no single causative mutation exists (75). Therefore, the most pressing challenge in the field is to extend therapeutic precision beyond monogenic targeting. This necessitates a dual strategy: first, advancing treatments that modulate convergent downstream pathologies (e.g., TDP-43 homeostasis, neuroinflammation) common across ALS forms; and second, developing biomarkers for meaningful stratification of the clinically and biologically heterogeneous sALS population. The future of ALS therapy lies in coupling the precision of genetic medicine with the breadth of mechanism-based treatments to benefit all patients.

Non-therapeutic interventions for ALS form the basis of a multifaceted care strategy aimed at improving and maintaining the quality of life of people with the disease. They range from the meticulous crafting of nutritional plans that cater to the unique dietary needs arising from ALS to the deployment of non-invasive ventilation techniques that ease respiratory distress, and the thoughtful application of physical therapy regimens that aim to maintain muscular function and forestall the onset of immobility. The inclusion of occupational and speech therapies, psychological support, and the timely introduction of palliative care, further enrich this comprehensive approach to disease management.

In summation, the advancement of ALS treatment is contingent upon a harmonized approach that converges pharmacological, genetic, and cellular therapies with a tapestry of supportive care. At present, the disease has not been cured, and its development cannot even be stopped. As the horizon of therapeutic possibilities expands, the imperative to navigate the disease’s heterogeneity and to forge personalized treatment strategies becomes ever more critical.
